# Wettability-Controlled Hydrophobic Coating of CMP Component Using PTFE and DLC for Mitigating Slurry Agglomeration and Contamination

**DOI:** 10.3390/mi16121382

**Published:** 2025-12-05

**Authors:** Eunseok Lee, Kyoungjun Sun, Yuhan So, Jaewoo Baek, Jun Hyuk Shin, Hae Dong Kim, Yeo Bin Youn, Min-Woo Kim

**Affiliations:** 1 Semiconductor Research Center, Research Line Technology Team, Samsung Electronics Co., Ltd., Hwaseong-si 18448, Republic of Korea; les1395@naver.com; 2 Division of Semiconductor Engineering, Myongji University, Yongin-si 17058, Republic of Korea; joon6389@naver.com (K.S.); syh7616@gmail.com (Y.S.); bjw9787@naver.com (J.B.); maplecake@naver.com (J.H.S.); cubedong17@naver.com (H.D.K.); yyb197x@naver.com (Y.B.Y.)

**Keywords:** CMP, hydrophobic coating, superhydrophobicity, slurry contamination, reliability

## Abstract

The chemical mechanical polishing (CMP) process in semiconductor fabrication faces challenges such as slurry agglomeration, scratches, and contamination, which degrade process reliability and device quality. To mitigate these challenges, this study investigated the application of hydrophobic surface coatings on CMP components. Polytetrafluorothylene (PTFE) was deposited onto stainless steel substrates, while diamond-like carbon (DLC) films were coated on PEEK-based retainer rings, with material selection guided by their surface energy characteristics and mechanical robustness. The hydrophobic performance of the coatings was systematically evaluated through contact angle measurements and surface roughness analysis (Ra, Rpk, Sa, Spk). Under oxide CMP conditions, 60 h reliability tests using non-patterned wafers demonstrated that PTFE-coated stainless-steel surfaces significantly reduced slurry-induced particle accumulation and suppressed scratches compared with uncoated substrates. In addition, PTFE provided stable hydrophobicity and effective scratch resistance, while DLC exhibited superhydrophobic behavior with contact angles exceeding 160°, offering potential for even greater protection against surface damage. The wettability of DLC coatings was further tunable via sp^3^/sp^2^ carbon bonding ratios and surface roughness, consistent with the predictions of the Cassie–Baxter and Wenzel models. These findings establish a framework for surface modification of CMP hardware. The integration of PTFE and DLC coatings effectively enhances hydrophobicity, suppresses slurry contamination, and improves scratch reliability, thereby offering a practical route for designing hydrophobic CMP components that strengthen process stability and extend equipment lifetime in advanced semiconductor manufacturing.

## 1. Introduction

Chemical mechanical polishing (CMP) is an essential process in semiconductor manufacturing, providing global planarization required for high-resolution photolithography and multi-level device integration [1]. By integrating controlled chemical reactions with mechanical abrasion, CMP enables nanometer-scale topographical control; in typical oxide and Cu CMP processes on 300 mm wafers, post-CMP wafer-scale non-planarity is commonly maintained within approximately 10–30 nm, with within-wafer non-uniformity below about 2–3% of the removed film thickness, while local step heights are controlled in the 1–5 nm range to satisfy advanced lithography requirements [2]. These capabilities are exploited across front-end and back-end processes, including shallow trench isolation (STI), poly-gate planarization, interlayer dielectric (ILD) formation, and copper metallization [1]. By integrating chemical surface reactions with nanometer-scale mechanical abrasion, CMP enables highly uniform removal of excess films and tight topographical control required in both front-end and back-end fabrication. Modern CMP processes routinely achieve global planarity on the order of 3–5 nm across a 300 mm wafer, with within-die surface variations controlled below 5 nm and defect dimensions such as scratch widths maintained in the 1–10 nm range, supporting reliable planarization for STI formation, poly-gate leveling, ILD gap-filling, and Cu metallization [2]. However, as device geometries continue to shrink and heterogeneous integration becomes mainstream, CMP reliability has emerged as a central bottleneck to achieving defect-free surfaces and maintaining high yield.

A persistent challenge arises from slurry–hardware interactions, where nanoscale abrasive particles, oxidizers, and stabilizers in the slurry interact with CMP components such as the retainer ring and membrane. These interactions often induce slurry agglomeration, particle adhesion, and non-uniform pressure distribution, ultimately leading to defects such as scratches, surface contamination, and particle redeposition [3,4]. Prior studies have explored defect origins including particle size distribution [5,6], pad surface residues [7], and scratch formation mechanisms [8], yet the contribution of hardware-induced defects has not been sufficiently addressed. In particular, irregularities at the hardware–pad–slurry interface exacerbate particle trapping and local mechanical stress, underlining the urgent need for surface modification strategies that directly target CMP component reliability.

Hydrophobic coatings provide a promising pathway to mitigate these limitations by reducing slurry adhesion and enhancing cleaning efficiency. Surface wettability, commonly described by Young’s relation, is governed by both surface roughness and surface free energy [9]. Higher apparent contact angles correspond to stronger water repellency, which effectively limits slurry adhesion. For rough surfaces, the Wenzel model accounts for liquid penetrating into surface asperities and amplifying the intrinsic wetting characteristics, whereas the Cassie–Baxter model describes droplets supported by air pockets trapped within the texture, which reduces the solid–liquid contact fraction and enhances hydrophobicity. In practice, however, these wetting states can be metastable: droplets initially in a Cassie–Baxter configuration may, under external perturbations such as pressure, vibration, or shear on tilted or moving surfaces, gradually transition toward a Wenzel-like state, leading to pronounced advancing–receding asymmetry and contact-angle hysteresis. Recent multiscale studies on the dynamic wettability of complex fractal isotropic surfaces have shown that such Cassie-to-Wenzel transitions are strongly coupled to surface topographic complexity and droplet sliding behavior [10,11,12].

Fluoropolymer coatings such as polytetrafluoroethylene (PTFE) and carbon-based films such as diamond-like carbon (DLC) have emerged as attractive candidates due to their intrinsically low surface energies, chemical stability, and mechanical robustness [13,14]. Nonetheless, most previous studies focused on static contact-angle measurements, with limited investigation into the coupling of roughness metrics (Ra, Rpk, Sa, Spk) with wettability and influence how it translates CMP scratch resistance during extended operation.

## 2. Experimental Section

### 2.1. Sample Preparation

To evaluate the performance of hydrophobic coatings under CMP conditions, two representative coating types were fabricated: Type A polytetrafluoroethylene (PTFE) and Type B diamond-like carbon (DLC) coatings. Each coating was deposited onto substrates commonly used in CMP component fabrication, namely stainless steel and polyetheretherketone (PEEK) to reflect realistic operating environments.

#### 2.1.1. Type A (PTFE Coatings)

PTFE, a fluoropolymer consisting of repeating –CF_2_–CF_2_– units with strong carbon-fluorine bonds, was deposited on stainless steel substrates via electrospraying or powder coating, followed by thermal curing at 350–400 °C. The resulting films exhibited exceptionally low surface energy, high chemical inertness, and non-adhesive characteristics, which effectively minimize slurry particle adhesion and facilitate post-process cleaning. Thermal curing also enhanced interfacial adhesion and mechanical robustness, ensuring stable hydrophobic performance during prolonged CMP exposure [15,16,17,18,19].

#### 2.1.2. Type B (DLC Coatings)

DLC films were deposited on PEEK substrates using physical vapor deposition (PVD) or plasma-enhanced chemical vapor deposition (PECVD). The film consists of mixed sp^2^/sp^3^ carbon hybridization within an amorphous matrix, which imparts hardness, wear resistance, and hydrophobic behavior.

In this study, the relative sp^3^/sp^2^ bonding ratio was qualitatively inferred from the PECVD/PVD deposition conditions, including substrate bias, ion energy, and hydrocarbon gas composition. Higher ion energy and substrate bias are known to promote sp^3^-rich bonding, whereas lower-energy deposition tends to favor sp^2^ hybridization. The moderate-energy conditions used in this work (80–200 °C) resulted in a mixed sp^3^/sp^2^ structure typical of amorphous DLC films.

Deposited at moderate temperatures (80–200 °C), the DLC coatings demonstrated long-term durability, high mechanical strength, and superior corrosion resistance, making them suitable for continuous CMP operation [20,21,22].

Table 1 summarizes the material composition, substrate selection, deposition methodology, thermal processing conditions, chemical structure, and surface properties. This comparison highlights the complementary suitability of each coating for different operational demands in CMP applications, ranging from chemical passivation to mechanical durability.

### 2.2. Contact Angle and Surface Roughness Evaluation

**Figure 1:**
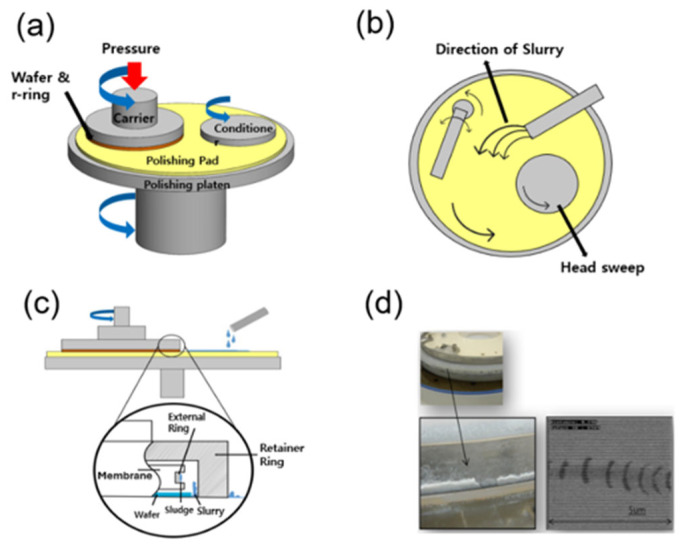
Schematic representation of the chemical mechanical polishing (CMP) process. (**a**) Basic configuration of the CMP system. (**b**) Slurry distribution and flow dynamics across the pad under head-sweeping motion. (**c**) Internal carrier structure highlighting potential slurry entrapment regions at the retainer and external rings. (**d**) Morphological evidence of slurry agglomeration and associated scratch defects observed on the wafer surface within the head region.

Figure 1 illustrates the CMP process, highlighting the dynamic interaction between the wafer, polishing pad, slurry, and carrier head. During planarization, nanoscale abrasive particles, typically silica-based, circulate within the slurry and repeatedly interact with the pad–wafer interface. The carrier head and retaining ring regulate local pressure distribution, but even subtle variations in flow and contact can lead to non-uniform slurry entrapment and defect formation. As the process progresses, agglomerated slurry particles may embed into the pad surface or adhere to hardware regions, initiating microscratches and residue buildup. These interfacial interactions form the basis for understanding how surface energy and wettability control are directly linked to CMP process reliability.

**Figure 2:**
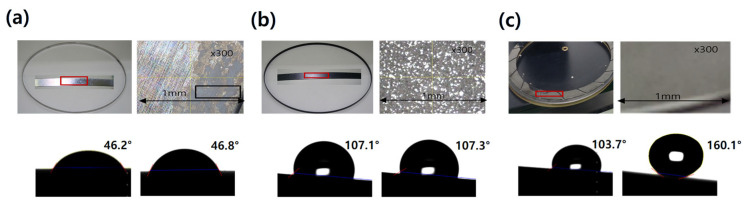
Surface characteristics of clamp rings before and after hydrophobic coating. (**a**) Uncoated stainless steel exhibiting slurry agglomeration with a low contact angle. (**b**) PTFE-coated sample (Type-A). (**c**) DLC-coated sample (Type-B).

Static water contact angles were measured using an SEO Phoenix MT analyzer (SEO Co. (Surface Electro Optics Co., Ltd.), Suwon, Republic of Korea) to evaluate hydrophobicity of each surface. Although the droplet images in Figure 2 may appear slightly inclined, this is caused by the automatic baseline leveling function of the KRÜSS DSA4 analysis software. All contact-angle measurements were performed on a leveled 0° horizontal surface; only the displayed baseline is visually corrected by the instrument. Each sample was measured at five distinct droplet positions, and the reported values represent their average. Both left and right contact angles were recorded for each droplet. The difference between the two values was consistently below 2°, well within the instrument’s repeatability range. Accordingly, the averaged static contact angle was used for reporting in Figure 2. A higher contact angle was interpreted as indicative of stronger water repellency and lower surface energy, consistent with the Cassie–Baxter and Wenzel wetting models. Surface morphology and topography were analyzed using a Keyence VK-X200 3D laser microscope (KEYENCE Corporation, Osaka, Japan). The roughness parameters analyzed included Ra (2D arithmetic average roughness), Rpk (2D reduced peak height), Sa (3D arithmetic mean height), and Spk (3D reduced summit height). Surface roughness parameters (Sa, Ra, Spk, Rpk) were obtained from five independent scan locations (707.74 μm × 530.71 μm each). Each measurement was performed over a 707 µm × 530 µm scan area. 3D surface profiling was conducted using a Keyence VK-X200 laser confocal microscope (KEYENCE Corporation, Osaka, Japan) with the following acquisition parameters: vertical resolution = 10 nm, lateral resolution = 0.5 μm, sampling interval = 0.5 μm, magnification = 50×, and a scan area of 707.74 μm × 530.71 μm. These settings ensure sufficient measurement resolution to accurately capture sub-micrometer surface features. Surface images and 3D roughness data were processed using the Keyence MultiFile Analyzer (KEYENCE Corporation, Osaka, Japan)/VK-H2X analysis software (KEYENCE Corporation, Osaka, Japan), which implements ISO 25178-based surface-parameter algorithms [23]. Among these, Spk was particularly emphasized as an indicator of air-pocket formation, which is critical for realizing superhydrophobic wetting behavior.

### 2.3. Scratch and Fouling Resistance Testing

Long-term reliability under CMP-like conditions was evaluated through a 60 h polishing test on non-patterned wafers (NPWs) using an oxide-based slurry. The wafers were polished on a Reflexion LK CMP system (Applied Materials, Santa Clara, CA, USA) equipped with standard IC-series polishing pads. Surface defects were quantified using a KLA Tencor SP2 inspection tool (KLA Corporation, Milpitas, CA, USA), with periodic of monitoring of scratch density. In addition, SEM imaging was employed to examine damaged regions and slurry-residue accumulation, enabling a detailed assessment of coating durability and fouling resistance during extended operation.

## 3. Results & Discussion

### 3.1. Macroscopic Response to Hydrophobic Coating

Building on this framework, Figure 2 compares clamp-ring surfaces before and after hydrophobic coating. By magnifying the clamp-ring region immediately after processing, slurry agglomeration and abrasive traces are clearly observed on uncoated stainless steel, suggesting that slurry residues readily compact at the hardware–pad–slurry interface. In contrast, the PTFE-coated surface maintained a smoother and cleaner surface under identical conditions, indicative of reduced adhesion and improved cleanability. Among the coated samples, the DLC surface showed the strongest repellency, with markedly suppressed contamination.

The quantitative sessile-drop measurements support these microscopic observations. As summarized, stainless steel [#1] exhibited a contact angle of 46.2°/46.8°, corresponding to a fully hydrophilic state. In contrast, PTFE [#2] and PEEK [#4] exhibited stable hydrophobic angles of approximately 107°, while DLC [#3] revealed a bimodal behavior with 103.7° and 160.1°, confirming the coexistence of partial and complete non-wetting regions. Plastic [#5] lay between these extremes with contact angles near 81°. The extremely high angle of 160.1° for DLC satisfies the classical criterion for superhydrophobicity, implying air-assisted droplet suspension at the solid–liquid interface. This transition from hydrophilic (steel) to hydrophobic (PTFE/PEEK) and superhydrophobic (DLC) states sets the stage for a more quantitative analysis of how texture influences wetting behavior.

### 3.2. Coordinated Texture–Wettability Correspondence

**Figure 3:**
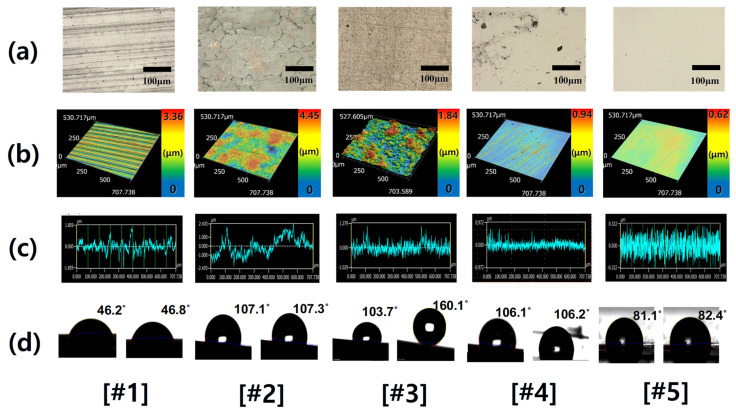
Comparative surface characterization of five representative samples [#1–5] under different coating conditions. (**a**) Optical microscope image (scale bar = 200 μm) reveals the overall surface morphology and distinguishes coating-dependent microstructural variations. (**b**) 3D laser profilometry over a 707.74 × 530.71 μm^2^ scan window highlights the spatial distribution of surface texture. (**c**) Roughness profiles along the *x*-axis (0–707.74 μm) illustrate height fluctuations associated with distinct coating macrotextures. (**d**) Contact angle measurements at different positions.

To rationalize the distinct wetting regimes observed macroscopically, the surface texture of the five samples was characterized using 3D laser profilometry and correlated with wettability data. Figure 3 presents representative 3D areal maps and X-direction height profiles acquired over a 707.7 × 530.7 µm window, revealing the hierarchical structures that govern droplet behavior. Corresponding roughness parameters (Ra, Rpk, Sa, Spk) are summarized in Table 2, where Ra and Sa describe the average 2D and 3D height variations, respectively. In contrast, the peak-related metrics Rpk and Spk quantify the protrusion of asperity summits above the core material zone and mean plane, allowing us to distinguish surfaces with tall, sparse peaks from those with more uniformly distributed summits. This combination of average and peak-sensitive parameters provides a quantitative basis for linking surface texture to hydrophobic and superhydrophobic responses.

The PTFE-coated surface exhibited a multiscale, non-uniform topography, combining large-scale undulations with fine asperities. Its elevated Ra (0.87 µm) and Sa (0.94 µm) reflect persistent areal roughness, while Spk remained moderate (~0.56 µm). This configuration stabilizes a hydrophobic wetting regime by enhancing surface–air interfaces without creating unstable summits. The observed contact angle (~107°) aligns with the Wenzel-type amplification of a low-energy fluorinated polymer.

In contrast, the DLC surface showed moderate Ra (0.27 µm) and Sa (0.38 µm) values but exhibited the highest Spk (1.10 µm) among all samples. This pronounced summit structure forms nanoscale cavities that trap air beneath the droplet, leading to localized non-wetting and the observed superhydrophobic state (160.1°). Stainless steel and plastic, with both low Sa and Spk (< 0.3 µm), exhibited weak hydrophobicity, while PEEK presented an intermediate case—Ra = 0.26 µm, Sa = 0.31 µm—with modest asperities sufficient to yield hydrophobic but not superhydrophobic behavior.

The roughness parameters are formally defined as follows:

2D Average Roughness [24]:

(1) $$Ra=\frac{1}{L}\int_{0}^{L}\left|z(x)\right|dx$$

3D Average Roughness [23]:

(2) $$Sa=\frac{1}{A}\iint_{A}\left|z(x,y)\right|dxdy$$

Here, Ra and Sa represent the t2D and 3D average roughness, respectively. Rpk is derived from the bearing area curve and reflects the height of asperities above the core material zone, whereas Spk denotes the summit height above the mean plane in 3D. Ra and Sa were selected to represent 2D and 3D mean roughness relevant to slurry spreading and liquid–solid interfacial behavior during CMP. Rpk and Spk quantify peak-dominant asperities and therefore capture the summit geometry that governs slurry adhesion and Cassie–Baxter air-pocket formation. In contrast, parameters such as Sz (maximum height) or Ssk (skewness) primarily describe extreme values or the shape of the height distribution, which are less sensitive to the peak-controlled wetting mechanisms relevant to PTFE- and DLC-coated surfaces.

**Figure 4:**
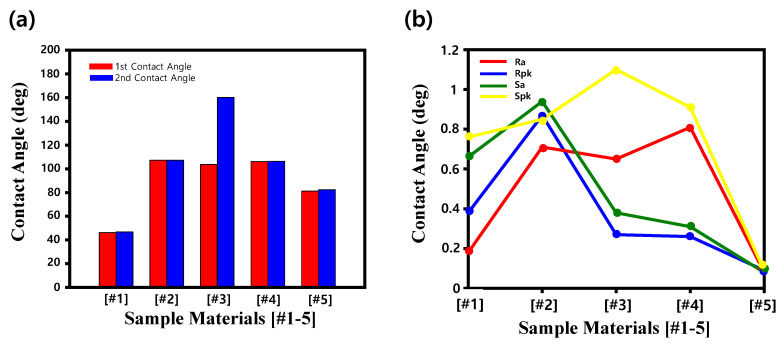
Quantitative evaluation of wettability and surface roughness for the five representative samples. (**a**) Contact angle measurement (^o^) at different positions after hydrophobic coating. (**b**) Comparative plot of roughness (Ra, Rpk, Sa, Spk) as a function of sample type, providing an integrated view of surface texture effects on hydrophobic behavior.

As illustrated in Figure 4, the interplay between Ra/Sa and Spk defines distinct hydrophobic regimes. PTFE leverages persistent areal roughness to maintain uniform hydrophobicity, whereas DLC combines low surface energy with high Spk to induce air-pocket-assisted superhydrophobicity. The remaining uncoated or polymeric samples, lacking these structural signatures, displayed weaker repellency. These findings collectively identify Spk and Sa as principal indicators of hydrophobic modulation, particularly when combined with low-surface-energy coatings such as PTFE and DLC. Such correlations provide an engineering basis for designing CMP components capable of minimizing slurry fouling and maintaining surface integrity under high-load planarization conditions.

### 3.3. Wetting Mechanism and Governing Equations

**Figure 5:**
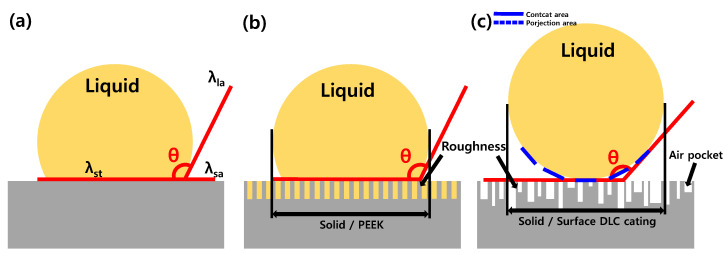
Schematic illustration of wetting behavior and air-pocket formation on coated surfaces, interpreted within the Cassie–Baxter framework. (**a**) Idealized smooth surfaces with direct liquid–solid contact, defining the intrinsic contact angle (θ). (**b**) Moderately textured PEEK surface, where roughness stabilized hydrophobicity without significant air entrapment. (**c**) Hierarchically rough DLC surface, where peak-dominated macrotextures promote air-pocket formation at the liquid–solid interface, enhanced hydrophobicity and superhydrophobic states.

The wettability trends established in Figure 2, Figure 3 and Figure 4 can be interpreted within classical wetting models. For an ideally smooth and chemically uniform surface, the droplet adopts the intrinsic contact angle *θ* (Figure 5a), according to Young’s equation [9]:

(3) $$cos\;\theta =\frac{\gamma_{S,A}-\;\gamma_{S,L}}{\gamma_{L,A}}$$

where *γ_S,A_*, *γ_S,L_*, and *γ_L,A_* denote the interfacial tensions at the solid–air, solid–liquid, and liquid–air boundaries, respectively. Real surfaces deviate from this ideal due to roughness-induced modification of the apparent contact angle θ*.

When liquid fully penetrates surface asperities, the apparent angle is described by the Wenzel relation [11],

(4) $$cos\;\theta^{*}=r\;cos\;\theta$$

with *r* representing the ratio of true to projected area. In this case (Figure 5b), roughness amplifies the intrinsic wettability of the substrate. This description is consistent with the PTFE case, where a fluorinated low-energy surface combined with elevated Ra and Sa, and moderate Spk stabilizes the hydrophobic state near 107°, reducing residue accumulation.

In contrast, surfaces with peak-dominated micro-textures can sustain air pockets, producing a Cassie–Baxter state (Figure 5c). Here, only a fraction $f_{s}$ of the nominal surface contacts the liquid and the apparent angle is described by [12]

(5) $$cos\;\theta^{*}=f_{s}\;(1+cos\;\theta )-1$$

where $f_{s}$ denotes the fraction of the liquid–solid contact area. A smaller $f_{s}\;$yields a higher apparent contact angle. The DLC coating, with its high Spk (≈1.10 µm) and moderate Sa (0.38 µm), clearly resides in this regime. The co-occurrence of contact angles at ~103.7° and 160.1° reflects partial transitions between Wenzel and Cassie–Baxter states, consistent with heterogeneous air entrapment. This duality arises from the distribution of summit heights that allow certain regions to remain wetted while others sustain air cushions.

A direct comparison of PEEK, PTFE, and DLC surfaces clarified the textural control of wettability. As summarized in Figure 4 and Table 2, PEEK displayed low Ra and Sa but relatively high Rpk (0.81 µm), producing isolated asperities insufficient for full air entrapment. The PTFE surface, characterized by hierarchical roughness (Ra ≈ 0.87 µm, Sa ≈ 0.94 µm), reinforces its low-energy fluorocarbon chemistry through a Wenzel-type mechanism [25]. DLC, with summit-dominated morphology, achieves Cassie–Baxter-type superhydrophobicity, confirming that fine control of Spk governs the air-pocket fraction and, consequently, wetting performance.

Collectively, these findings demonstrate that surface roughness and chemical composition act synergistically to tune wettability. PTFE stabilizes hydrophobic behavior by extending low-energy domains over a roughened area, while DLC achieves superhydrophobicity through discrete high-asperity structures. PEEK, lacking either condition, remains in a stable but moderate hydrophobic regime.

This description is consistent with the DLC surface, where the highest Spk (Spk ≈ 1.10 µm) combined with moderate Sa produced localized air entrapment, yielding a superhydrophobic contact angle of 160.1°. The observation of dual contact angles (~103.7° and 160.1°) further suggests that different regions on the DLC surface locally oscillate between Wenzel-type penetration and air-assisted non-wetting. This heterogeneity originates from the distribution of summit heights (Spk ≈ 1.10 µm) and moderate Sa (0.38 µm), which together dictate local wetting states.

A direct comparison of PEEK, PTFE, and DLC surfaces clarifies how distinct roughness parameters govern hydrophobic performance under CMP conditions. As shown in Figure 4 and consolidated in Table 2, the PEEK surface exhibited relatively low roughness (Ra = 0.26 μm, Sa = 0.31 μm) but a comparatively high Rpk value (0.81 μm), indicating isolated asperity peaks without a broad supporting relief. The measured contact angle (~106°) confirmed a hydrophobic but not superhydrophobic state, with limited ability to sustain air entrapment due to modest summit feature (Spk = 0.91 μm). These findings emphasize that Spk and Sa are critical parameters governing air-pocket formation and contact angle enhancement, and highlight the potential of the DLC coating for achieving robust superhydrophobicity, suppressing slurry fouling, and improving CMP reliability.

These texture–wettability correlations are summarized in Table 3, which integrates Spk-dominated peak morphology, measured contact angles, and the resulting hydrophobic/superhydrophobic wetting regimes for each surface.

### 3.4. Reliability Under NPW Oxide-CMP

**Figure 6:**
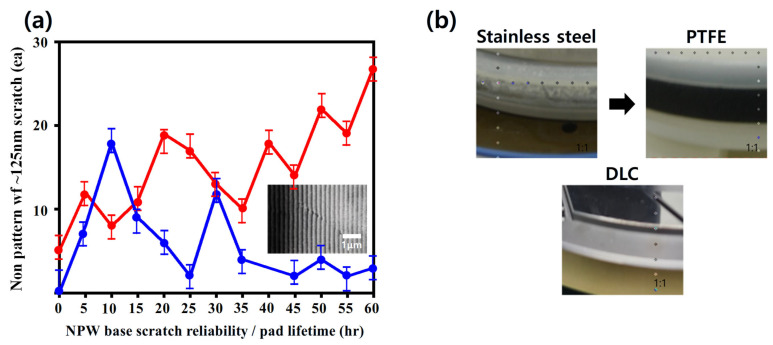
Reliability evaluation of hydrophobic coating under oxide CMP conditions. (**a**) Scratch count evolution on non-pattern wafers (NPWs) under CMP reliability testing with oxide slurry for uncoated stainless-steel clamp ring (red curve) and PTFE-coated sample (blue curve). Inset: representative SEM image of a scratch (~5 μm scale bar). (**b**) Microscopic clamp-ring surface after CMP exposure for stainless surface, PTFE-coated surface, and DLC-coated surface.

To evaluate long-term stability and functional performance under realistic CMP conditions, a 60 h reliability test was conducted using non-patterned wafers (NPWs) and oxide-based slurry. The experimental setup replicated industrial planarization parameters, with continuous slurry circulation, pad rotation, and periodic rinsing cycles to simulate extended operation. Figure 6a presents the time-dependent scratch count comparison between bare stainless steel and the PTFE-coated (Type-A) sample. The PTFE-coated sample (Type-A) consistently suppressed scratch formation compared to bare stainless steel, achieving 30% fewer scratches after 60 h. This reduction indicates that the PTFE layer not only enhances initial surface integrity but also provides sustained protection during extended polishing. The plot reports the absolute number of scratches counted over the wafer area at each 5 h interval, and standard-deviation error bars are included (*n* = 3 wafers per condition) to indicate statistical variability.

Figure 6b provides the post-CMP surface images used for quantifying fouling behavior. Fouling resistance was evaluated by measuring the areal coverage of adhered slurry residue after 60 h CMP. The optical images were converted to grayscale, threshold-segmented, and analyzed to extract the residue-covered fraction. The PTFE-coated clamp ring exhibited a residue coverage of 12.4%, whereas the uncoated stainless-steel ring showed 38.7%. This substantial reduction in residue accumulation confirms the fouling-mitigation capability of the PTFE coating under extended CMP operation.

To further assess the long-term stability of the coating, the surface properties of the clamp ring were re-measured after the 60 h CMP test. The PTFE coating exhibited excellent long-term stability, with the static contact angle decreasing only slightly from 107.3° to 105.8° (Δ = 1.5°). In addition, the key roughness parameters (Sa and Spk) remained within ±0.02 μm of their initial values, indicating that neither the hierarchical microtexture nor the summit-dominated asperity structure degraded during prolonged polishing. These results collectively confirm that the PTFE coating maintains both hydrophobic functionality and mechanical integrity under long-term CMP loading.

Furthermore, the durability of both coatings under chemical and thermal stress was confirmed by post-test EDS and morphological analyses, which showed no evidence of fluorine depletion or carbon oxidation. This robustness suggests that both PTFE and DLC coatings can endure extended CMP environments without degradation, providing a sustainable route to improving tool lifetime and planarization reliability.

## 4. Conclusions

In this study, the hydrophobic behavior of PTFE- and DLC-coated CMP components was systematically investigated by correlating contact angle, surface roughness parameters (Ra, Rpk, Sa, Spk), and surface energy. Both coatings exhibited markedly enhanced water repellency compared with uncoated stainless steel.

The PTFE coating, stabilized by strong C–F bonding and a hierarchical texture, achieved a consistent contact angle of ~107°, consistent with the Wenzel and Owens–Wendt models. In contrast, the DLC coating, defined by an sp^3^/sp^2^ carbon structure and a pronounced summit roughness (Spk = 1.1 µm), reached a superhydrophobic angle of 160.1°, consistent with the Cassie–Baxter wetting regime. Surface roughness emerged as a critical factor, with PTFE benefiting from elevated Ra values, while DLC relied on peak-dominated Spk features to sustain air-pocket-assisted non-wetting. PEEK, despite its inherently low surface energy, exhibited only moderate hydrophobicity due to structural limitations.

Reliability testing confirmed that PTFE reduced defect counts by approximately 30% compared with uncoated stainless steel, demonstrating its effectiveness in suppressing contamination and wear. The DLC coating, with its superhydrophobic behavior, further promises effective suppression of slurry adhesion and frictional wear, highlighting its potential for long-term durability. Taken together, these findings demonstrate that coupling wettability control with tailored roughness design offers a robust pathway toward more reliable and cleaner CMP hardware. This approach provides a practical framework for engineering advanced component coatings that mitigate fouling, reduce defects, and enhance process stability in next-generation semiconductor manufacturing.

## Figures and Tables

**Table 1:** Comparison of Type A PTFE and Type B coatings in terms of material composition, processing condition, and surface properties.

Property	Type-A PTFE Coating	Type-B DLC Coating
Material	Polytetrafluoroethylene	Diamond like carbon
Principle	Low coefficient of friction and chemical	High hardness, wear resistance
Manufacturing method	Spray, powder coating	PVD, CVD, ion beam deposition
Temperature	350–400 °C	80–200 °C
Substance	C_2_F_4_ polymer chains with strong –CF_2_–CF_2_– bonding	Amorphous C–H bonding with sp^3^/sp^2^ hybridization
Chemical formula	(C_2_F_4_)_n_/–CF_2_–CF_2_	C (sp^3^/sp^2^) network
Manufacturer	Not disclosed	Not disclosed
Molecular weight	Avg. polymer: ~10^6^ g/mol	Not defined, variable depending
Surface Properties	Non-adhesive, hydrophobic, chemically resistant	High wear resistance, low friction, corrosion resistant

**Table 2:** Surface roughness parameters of representative CMP component materials before and after hydrophobic coating.

Sample Test	Ra	Rpk	Sa	Spk
Stainless [#1]	0.38	0.18	0.66	0.76
PTFE coated Type A [#2]	0.87	0.71	0.94	0.85
DLC coated Type B [#3]	0.27	0.65	0.38	1.1
PEEK [#4]	0.26	0.81	0.31	0.91
Plastic [#5]	0.09	0.08	0.08	0.09

**Table 3:** Summary of wettability behavior of coated surface (DLC, PTFE, PEEK) under CMP conditions. The table compares surface roughness (Spk), measured contact angles (°), and the dominant hydrophobic mechanisms governing wetting states.

Material	Surface Roughness (Spk)	Contact Angle (θ)	Hydrophobic Mechanism	Hydrophobic
DLC	1.1	160.1	Cassie-Baxter (f ≈ 0.08)	Super hydro phobic
PTFE	0.85	107.3	Wenzel (r ≈ 1.1)	Ultra hydrophobic
PEEK	0.91	106.2	Low γ_s_^p^ (≈15 mN/m)	Hydrophobic

## Data Availability

The original contributions presented in the study are included in the article, further inquiries can be directed to the corresponding author.
